# Effects of a Sativex-Like Combination of Phytocannabinoids on Disease Progression in R6/2 Mice, an Experimental Model of Huntington’s Disease

**DOI:** 10.3390/ijms18040684

**Published:** 2017-03-23

**Authors:** Sara Valdeolivas, Onintza Sagredo, Mercedes Delgado, Miguel A. Pozo, Javier Fernández-Ruiz

**Affiliations:** 1 Departamento de Bioquímica y Biología Molecular, Instituto Universitario de Investigación en Neuroquímica, Facultad de Medicina, Universidad Complutense, 28040 Madrid, Spain; svaldeolivas@med.ucm.es; 2 Centro de Investigación Biomédica en Red de Enfermedades Neurodegenerativas (CIBERNED), 28040 Madrid, Spain; 3 Instituto Ramón y Cajal de Investigación Sanitaria (IRYCIS), 28040 Madrid, Spain; 4 Unidad de Cartografía Cerebral, Instituto Pluridisciplinar, Universidad Complutense, 28040 Madrid, Spain; mwallace@pluri.ucm.es (M.D.); pozo@med.ucm.es (M.A.P.); 5 Departamento de Farmacología, Facultad de Farmacia, Universidad Complutense, 28040 Madrid, Spain; 6 Departamento de Fisiología, Facultad de Medicina, Universidad Complutense, 28040 Madrid, Spain

**Keywords:** phytocannabinoids, cannabidiol, Δ^9^-tetrahydrocannabinol, Huntington’s disease, R6/2 mice, basal ganglia, neuroprotection

## Abstract

Several cannabinoids afforded neuroprotection in experimental models of Huntington’s disease (HD). We investigated whether a 1:1 combination of botanical extracts enriched in either ∆^9^-tetrahydrocannabinol (∆^9^-THC) or cannabidiol (CBD), which are the main constituents of the cannabis-based medicine Sativex^®^, is beneficial in R6/2 mice (a transgenic model of HD), as it was previously shown to have positive effects in neurotoxin-based models of HD. We recorded the progression of neurological deficits and the extent of striatal deterioration, using behavioral, in vivo imaging, and biochemical methods in R6/2 mice and their corresponding wild-type mice. The mice were daily treated, starting at 4 weeks after birth, with a Sativex-like combination of phytocannabinoids (equivalent to 3 mg/kg weight of pure CBD + ∆^9^-THC) or vehicle. R6/2 mice exhibited the characteristic deterioration in rotarod performance that initiated at 6 weeks and progressed up to 10 weeks, and elevated clasping behavior reflecting dystonia. Treatment with the Sativex-like combination of phytocannabinoids did not recover rotarod performance, but markedly attenuated clasping behavior. The in vivo positron emission tomography (PET) analysis of R6/2 animals at 10 weeks revealed a reduced metabolic activity in the basal ganglia, which was partially attenuated by treatment with the Sativex-like combination of phytocannabinoids. Proton nuclear magnetic resonance spectroscopy (H^+^-MRS) analysis of the ex vivo striatum of R6/2 mice at 12 weeks revealed changes in various prognostic markers reflecting events typically found in HD patients and animal models, such as energy failure, mitochondrial dysfunction, and excitotoxicity. Some of these changes (taurine/creatine, taurine/*N*-acetylaspartate, and *N*-acetylaspartate/choline ratios) were completely reversed by treatment with the Sativex-like combination of phytocannabinoids. A Sativex-like combination of phytocannabinoids administered to R6/2 mice at the onset of motor symptoms produced certain benefits on the progression of striatal deterioration in these mice, which supports the interest of this cannabinoid-based medicine for the treatment of disease progression in HD patients.

## 1. Introduction

Huntington’s disease (HD) is an inherited neurodegenerative disorder characterized by motor alterations (chorea followed by akinesia), but also cognitive dysfunction and psychiatric symptoms [1]. HD is primarily caused by a mutation in the huntingtin gene consisting of a CAG triplet repeat expansion translated into an abnormal polyglutamine tract in the N-terminus of this protein, which becomes toxic for striatal and cortical neuronal subpopulations [2]. The available pharmacotherapy to alleviate HD symptoms is poor (e.g., tetrabenazine for chorea), whereas there are no available treatments able to arrest/delay disease progression in HD. In recent years, several compounds (e.g., minocycline, coenzyme Q10, unsaturated fatty acids, inhibitors of histone deacetylases) have been investigated and produced encouraging effects in preclinical studies, but none of the findings obtained in these studies have yet led to the development of an effective medicine [3].

Continuing on from an extensive preclinical evaluation using different experimental models of HD, clinical tests are now being performed with cannabinoids [4]. Such preclinical evaluation demonstrated preservation of striatal neurons by several cannabinoid agonists against different cytotoxic stimuli that operate in HD pathogenesis [5,6]. These beneficial effects were exerted through multiple mechanisms of action, some of which involve the activation of CB_1_ and/or CB_2_ receptors and others of which do not. For example, cannabinoids with an antioxidant profile (i.e., ∆^9^-tetrahydrocannabinol (∆^9^-THC), cannabidiol (CBD), or cannabigerol (CBG)) protected striatal neurons against oxidative injury caused by the mitochondrial complex II inhibitor 3-nitropropionic acid (3NP), and this occurred through effects that were CB_1_/CB_2_ receptor-independent [7,8,9]. Given its activity at the CB_1_ and CB_2_ receptors, ∆^9^-THC was also investigated with positive results in other experimental models, for example, R6/2 mice, a transgenic mouse model of HD [10]. The effects of ∆^9^-THC in these mice are likely produced through the activation of CB_1_ receptors [10], but they could also involve the activation of CB_2_ receptors, as selective agonists of this receptor type preserved striatal neurons in this genetic model [11] and also in malonate-lesioned rats [12], a model priming apoptotic death and glia-driven inflammation. Selective agonists of CB_1_ [10,13] and CB_2_ [11] receptors also preserved striatal neurons in in vitro or in vivo excitotoxic models, whereas other authors did not find any beneficial effect in R6/1 mice using ∆^9^-THC, the synthetic agonist HU-210, and the inhibitor of the endocannabinoid metabolism URB597 [14].

The data collected from these studies support the interest of evaluating cannabinoids as disease modifiers in HD in patients, but they also suggest that this should be done with a broad-spectrum cannabinoid. As an alternative, a combination of cannabinoids having complementary pharmacological effects would also be adequate. Such a profile can be found in Sativex, a cannabis-based medicine which has been licensed for alleviating specific symptoms (spasticity, pain) in patients affected by multiple sclerosis [15,16]. ∆^9^-THC present in Sativex may activate CB_1_/CB_2_ receptors and exert receptor-independent antioxidant effects, which would be markedly enhanced by CBD also present in Sativex [17]. A few years ago, we initiated experiments with the Sativex-like combination of these two phytocannabinoids in different animal models of HD. We used those models in which individual cannabinoids had been active [7,8,10,11,12,13]. We worked first with rats subjected to 3NP intoxication [18], in which calpain activation and oxidative injury are important cytotoxic mechanisms. In this model, the administration of pure ∆^9^-THC [7] or pure CBD [8] displayed neuroprotective properties. We found that, using the Sativex-like combination of these two phytocannabinoids, striatal neurons were also preserved against the 3NP intoxication and, again, we found that such a neuroprotective effect was CB_1_/CB_2_ receptor independent [18]. Next, we conducted similar studies in a second model of HD, rats unilaterally-lesioned with malonate, in which activation of the apoptotic machinery and glial activation/inflammatory events were responsible for the striatal damage. We had previously described that selectively activating the CB_2_ receptor resulted in a reduction of the striatal damage [12], whereas this was aggravated after CB_1_ receptor blockade [19]. The Sativex-like combination of phytocannabinoids also preserved striatal neurons from death caused by malonate, and this effect was dependent on both CB_1_ and CB_2_ receptors [20].

These positive effects prompted us to extend our research to transgenic mouse models of HD, specifically the R6/2 mice, which are frequently used for the evaluation of potential neuroprotective compounds that warrant investigation at the clinical level. To this end, we subjected R6/2 mice to daily treatment with Sativex-like combination of phytocannabinoids (equivalent to 3 mg/kg weight of pure CBD + ∆^9^-THC) or with vehicle. Treatments commenced at 4 weeks and were prolonged up to 12 weeks after birth, the age at which animals were euthanized. The progression of neurological deficits (e.g., rotarod performance) was recorded during the treatment period, whereas additional motor markers (e.g., clasping behavior) were recorded just before animals were euthanized together with the in vivo imaging analysis of local metabolic activity using positron emission tomography (PET). Once euthanized, brains were collected and the striatum dissected and used for ex vivo proton magnetic resonance spectroscopy (H^+^-MRS) analysis, which provides a series of detectable biomarkers reflecting: (i) oxidative damage (e.g., reduced glutation/creatine ratio (GSH/Cre)); (ii) energy failure (e.g., elevated lactate/*N*-acetyl-aspartate ratio (Lac/NAA), reduced NAA/choline ratio (NAA/Cho)); (iii) excitotoxicity (e.g., increased glutamate/NAA ratio (Glu/NAA)); and (iv) other events typically found in HD (e.g., taurine/Cre ratio (Tau/Cre), Tau/NAA ratio, all of them being relatively prognostic for brain integrity/damage) [21,22,23,24,25,26].

## 2. Results

### 2.1. Behavioral Analysis of R6/2 Mice Treated with the Sativex-Like Combination of Phytocannabinoids

**Figure 1:**
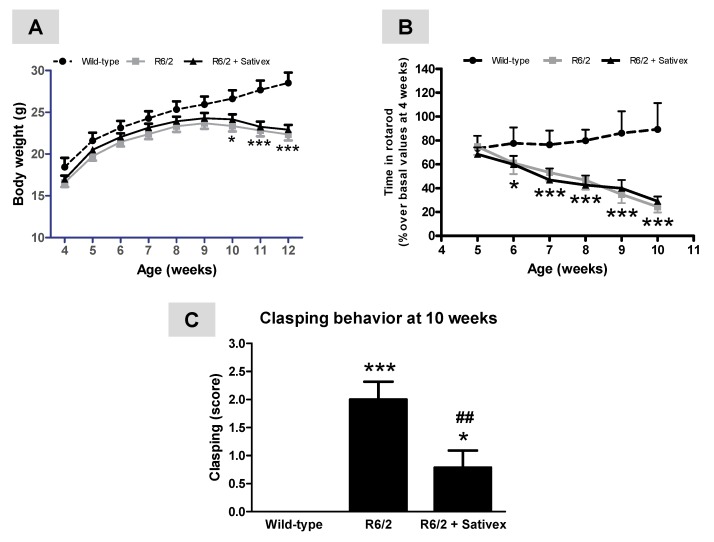
Weight gain (**A**), rotarod performance (**B**), and clasping behavior (**C**) in R6/2 mice treated from the age of 4 weeks after birth with ∆^9^-tetrahydrocannabinol (∆^9^-THC)- and cannabidiol (CBD)-enriched botanical extracts combined in a Sativex^®^-like ratio 1:1 (4.5 mg/kg equivalent to 3 mg/kg of pure CBD + ∆^9^-THC), or vehicle (Tween 80-saline), and the corresponding wild-type animals. Details in the text. Values are expressed as means ± SEM for six to eight animals per group. Data were subjected to one- (clasping) or two-way (rotarod and weight) analysis of variance followed by the Student-Newman-Keuls test (* *p* < 0.05, *** *p* < 0.005 compared with wild-type mice; ^##^ *p* < 0.01 compared to R6/2 mice treated with vehicle).

Despite the possibility of differences by gender in disease progression and treatment responses in R6/2 mice, our experiment was conducted exclusively in males to avoid a potential influence of ovarian steroid fluctuations when using females. Compared to wild-type animals, R6/2 mice exhibited a characteristic loss of weight gain that was initiated at 9 weeks of age and deteriorated at 10–11 weeks and up to 12 weeks of age when animals were euthanized (age: F(8,200) = 279.1, *p* < 0.0001; genotype/treatment: F(2,200) = 4.497, *p* < 0.05; interaction: F(16,200) = 10.64, *p* < 0.0001; Figure 1A). This loss of weight has been widely reported in R6/2 mice [9,10] and also in other transgenic models of HD [27,28]. In parallel, there was a deterioration in rotarod performance that was already evident at the age of 6 weeks after birth, then occurring before the loss of weight, and reaching its maximum at the last age analyzed (10 weeks after birth) (age: F(5,139) = 21.57, *p* < 0.0001; genotype/treatment: F(2,139) = 187.3, *p* < 0.0001; interaction: F(10,139) = 15.30, *p* < 0.0001; Figure 1B). The treatment with the Sativex-like combination of phytocannabinoids did not produce any recovery in both rotarod performance and weight gain (Figure 1A,B). By contrast, the Sativex-like combination of phytocannabinoids was highly effective in attenuating clasping behavior detected in R6/2 mice at the age of 10 weeks (F(2,17) = 13.88, *p* < 0.0005; <95% confidence intervals for wild-type mice, and R6/2 animals treated with vehicle or Sativex-like combination of phytocannabinoids were 0, 1.12. and 0.04, respectively; >95% confidence intervals were 0, 2.88, and 1.53, respectively; Figure 1C), a response that reflects dystonia and is absent in wild-type animals. The statistical relevance of these differences was confirmed using different post-hoc assays (Student-Newman-Keuls, Tukey, and Bonferroni), all reaching similar statistics.

### 2.2. In Vivo Imaging Analysis of Regional Brain Metabolic Activity in R6/2 Mice Treated with the Sativex-Like Combination of Phytocannabinoids

**Figure 2:**
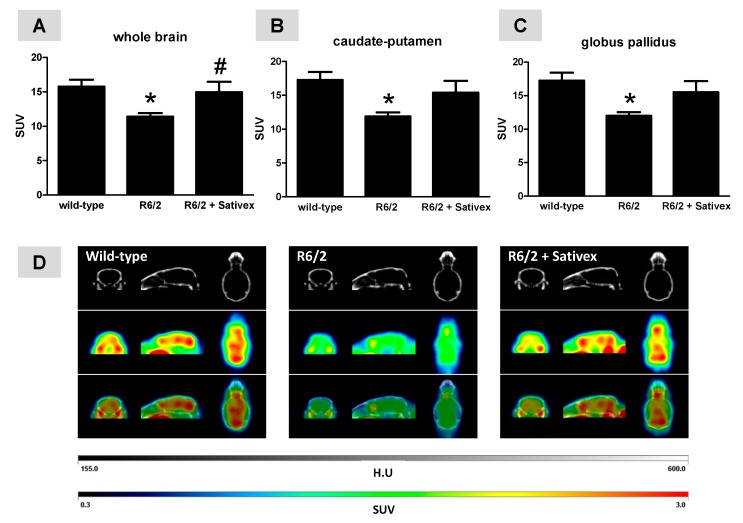
In vivo glucose metabolic rates measured by positron emission tomography (PET) analysis in the whole brain (**A**), caudate-putamen (**B**), and globus pallidus (**C**), including representative PET images in (**D**) of R6/2 mice (at 10 weeks after birth) treated from the age of 4 weeks after birth with ∆^9^-THC- and CBD-enriched botanical extracts combined in a Sativex^®^-like ratio 1:1 (4.5 mg/kg equivalent to 3 mg/kg of pure CBD + ∆^9^-THC), or vehicle (Tween 80-saline), and the corresponding wild-type animals. Details in the text. Values correspond to standard uptake value (SUV) and are expressed as means ± SEM of five subjects per group and age. Data were subjected to one-way analysis of variance followed by the Student-Newman-Keuls test (* *p* < 0.05 compared with wild-type mice; ^#^ *p* < 0.05 compared to R6/2 mice treated with vehicle).

The PET analysis with [^18^F]-fluoro-deoxy-glucose ([^18^F]FDG) of R6/2 mice proved a reduced metabolic activity in the whole brain at 10 weeks (F(2,14) = 4.609, *p* < 0.05; Figure 2A). Such reduction was also evident in those brain areas more affected in R6/2 mice such as the caudate-putamen (F(2,14) = 4.593, *p* < 0.05; Figure 2B) and the globus pallidus (F(2,14) = 4.794, *p* < 0.05; Figure 2C), but it was also present in the cerebral cortex, cerebellum, amygdala, hypothalamus, and other forebrain regions (data not shown). In all cases, the reduction in regional metabolic activity in the R6/2 mice was attenuated by treatment with the Sativex-like combination of phytocannabinoids (Figure 2A–C). In the case of the whole brain, the difference between R6/2 mice treated with vehicle and treated with the Sativex-like combination of phytocannabinoids reached statistical significance (<95% confidence intervals for wild-type mice, and R6/2 animals treated with vehicle or Sativex-like combination of phytocannabinoids were 12.99, 10.01, and 10.79, respectively; >95% confidence intervals were 18.57, 12.80, and 19.12, respectively; Figure 2A), but in the case of the caudate-putamen and globus pallidus, the effect was evident only in the loss of statistical significance when compared to wild-type animals (caudate-putamen: <95% confidence intervals for wild-type mice, and R6/2 animals treated with vehicle or Sativex-like combination of phytocannabinoids were 13.95, 10.19, and 10.52, respectively; >95% confidence intervals were 20.56, 13.56, and 20.27, respectively; Figure 2B; globus pallidus: <95% confidence intervals for wild-type mice, and R6/2 animals treated with vehicle or Sativex-like combination of phytocannabinoids were 13.98, 10.48, and 10.86, respectively; >95% confidence intervals were 20.53, 13.51, and 20.14, respectively; Figure 2C). Effects similar to the caudate-putamen and the globus pallidus by the treatment with the Sativex-like combination of phytocannabinoids were also evident in the cerebellum (data not shown), other Central Nervous System (CNS) structure that has been related to the control of motor activity. In other brain regions (e.g., septum nuclei, amygdala) the differences between R6/2 mice treated with vehicle or with the Sativex-like combination of phytocannabinoids were statistically significant (data not shown), as for the whole brain, but these regions are not related to the control of movement but to emotional and cognitive processes which, although also affected in HD patients, were not reproduced in R6/2 mice. Representative PET images for the three groups investigated are included in Figure 2D.

### 2.3. H^+^-MRS Analysis of Several Markers of Brain Integrity in R6/2 Mice Treated with the Sativex-Like Combination of Phytocannabinoids

**Figure 3:**
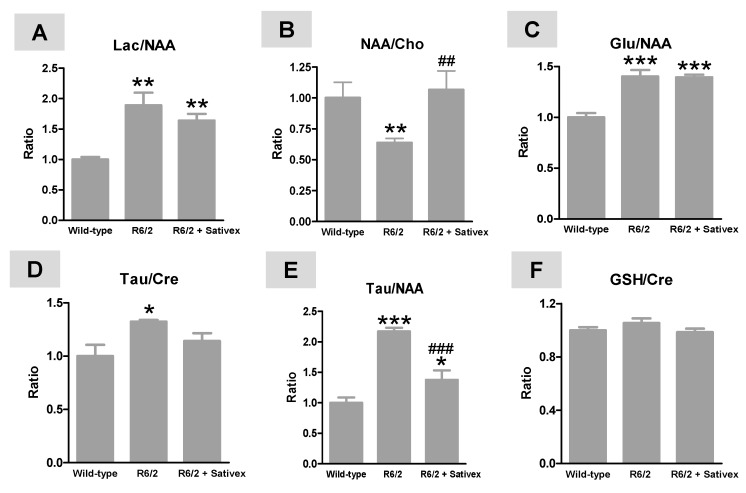
Lactate/*N*-acetyl-aspartate (Lac/NAA) (**A**); NAA/ choline (Cho) (**B**); glutamate (Glu)/NAA (**C**); Tau/Cre (**D**); Tau/NAA (taurine/creatine) (**E**); and (reduced glutation) GSH/Cre (**F**) ratios measured by proton nuclear magnetic resonance spectroscopy (H^+^-MRS) analysis in the striatum of R6/2 mice (at 12 weeks after birth) treated from the age of 4 weeks after birth with ∆^9^-THC- and CBD-enriched botanical extracts combined in a Sativex^®^-like ratio 1:1 (4.5 mg/kg equivalent to 3 mg/kg of pure CBD + ∆^9^-THC), or vehicle (Tween 80-saline), and the corresponding wild-type animals. Details in the text. Values are expressed as means ± SEM of five animals per group. Data were subjected to one-way analysis of variance followed by the Student-Newman-Keuls test (* *p* < 0.05, ** *p* < 0.01, *** *p* < 0.005 compared with wild-type mice; ^##^ *p* < 0.01, ^###^ *p* < 0.005 compared to R6/2 mice treated with vehicle).

The H^+^-MRS analysis of the post-mortem striatum of R6/2 animals at 10 weeks after birth demonstrated important changes in specific markers of HD pathology. For example, we found an elevation in Lac/NAA ratio (F(2,14) = 10.99, *p* < 0.005; Figure 3A), which is an indirect marker of a possible energy deficit and mitochondrial dysfunction due to elevated Lac generation, a result also found by other authors [24,25], and/or reduced NAA levels possibly reflecting neuronal dysfunction/damage [24]. Lowered NAA levels also may reflect mitochondrial dysfunction, as this metabolite is synthesized in mitochondria [24]. This elevation tended to be reduced, although modestly (without reaching statistical significance) by the treatment with the Sativex-like combination of phytocannabinoids (Figure 3A). However, this treatment completely recovered the reduction in NAA/Cho ratio found in R6/2 mice (F(2,12) = 11.62, *p* < 0.005; (<95% confidence intervals for wild-type mice, and R6/2 animals treated with vehicle or Sativex-like combination of phytocannabinoids were 0.84, 0.55, and 0.88, respectively; >95% confidence intervals were 1.16, 0.72, and 1.26, respectively; Figure 3B), which is also a potential predictive marker of energy failure [24,25]. We also found an elevation in Glu/NAA ratio (F(2,14) = 24.81, *p* < 0.0001), which potentially reflects excitotoxicity [21,22,23,26], but this response was not altered by the treatment with the Sativex-like combination of phytocannabinoids (Figure 3C). The H^+^-MRS analysis also revealed elevated Tau/Cre (F(2,14) = 4.572, *p* < 0.05; Figure 3D) and Tau/NAA (F(2,14) = 30.08, *p* < 0.0001; Figure 3E) ratios in R6/2 mice. Elevated Tau levels—presumably reflecting an endogenous protective response, as well as changes in cell volume regulation, and being frequently associated with lower Cre concentrations potentially reflecting reduced energy availability—have been found in HD patients using metabolomic analysis [29] and in other mouse models of HD [26]. These changes were attenuated by the treatment with the Sativex-like combination of phytocannabinoids, in particular for Tau/NAA ratio (<95% confidence intervals for wild-type mice, and R6/2 animals treated with vehicle or Sativex-like combination of phytocannabinoids were 0.76, 2.00, and 0.94, respectively; >95% confidence intervals were 1.24, 2.34, and 1.81, respectively; Figure 3E) and, to a lesser extent (only losing statistical significance with respect to wild-type mice), for Tau/Cre ratio (<95% confidence intervals for wild-type mice, and R6/2 animals treated with vehicle or Sativex-like combination of phytocannabinoids were 0.70, 1.28, and 0.93, respectively; >95% confidence intervals were 1.30, 1.37, and 1.35, respectively; Figure 3D). No changes were noted in GSH/Cre ratio (F(2,14) = 1.583, ns), potentially reflecting oxidative damage [30], in R6/2 mice compared to wild-type animals, which agrees with the poor oxidative stress response described in these mice [9]; treatment with the Sativex-like combination of phytocannabinoids did not modify this ratio (Figure 3F).

## 3. Discussion

Only a few clinical studies have been performed to determine whether cannabinoid compounds have beneficial effects in HD [31,32,33,34]. These clinical studies concentrated more on HD symptoms (e.g., choreic movements, behavioral disturbances) rather than on disease progression. Recent animal studies, however, have demonstrated that using a broad-spectrum cannabinoid or, alternatively, combinations of cannabinoids having complementary profiles, it is possible to delay the progression of the disease and to preserve the integrity of striatal neurons, and this has been found in different animal models of HD [8,9,10,11,12,13]. Such observations demand new clinical studies directed at testing in patients these disease-modifying properties demonstrated by certain cannabinoids. To provide more support to this idea, we recently demonstrated neuroprotective effects, using the Sativex-like combination of ∆^9^-THC and CBD botanical extracts, in rats subjected to 3NP intoxication [18] and also in malonate-lesioned rats [20], two classic experimental in vivo models of HD in which striatal damage is caused by different primary mechanisms (e.g., calpain activation/oxidative injury, apoptosis/gial activation/inflammation, respectively).

In the present study, we used R6/2 mice, a genetic model which is frequently used in the preclinical evaluation of novel therapies for HD because of the rapid disease progression of these animals, and in which different cannabinoids administered individually, including ∆^9^-THC but not CBD, were shown to be active [10,11]. Our results suggest certain benefits with the Sativex-like combination of phytocannabinoids in R6/2 mice in line with those observed in neurotoxin-based models [18,20]. In our hands, the mixture of both phytocannabinoids attenuated clasping behavior, which represents a classic motor symptom found in these mice, although it was inactive against the progressive worsening in rotarod performance. This lack of Sativex benefits in mouse rotarod performance was relatively surprising, as we have previously found that this parameter, being markedly reduced in R6/2 mice, was attenuated by the treatment with pure ∆^9^-THC [10]. In our opinion, it is not a problem of insufficient dose, as we have preliminary and unpublished data indicating that higher doses (5 and 10 mg/kg) for the Sativex-like combination of phytocannabinoids led to the same lack of improvement. We do not consider that this improvement may be elicited by increasing the number of subjects in both experimental groups, as they were relatively homogeneous with their progressing lines for the rotarod performance showing a high overlapping. In order to find an explanation it is better to look at other studies that have also described no improvement in rotarod performance but beneficial effects in other behavioral parameters and recovery at the neuropathological level. This was the case of our previous study with the phytocannabinoid CBG, which proved a poor and statistically non-significant recovery in the worsened rotarod response typical of R6/2 mice, but it produced a much more marked reduction in the presence of mutant huntingtin aggregates as well as an increase in different prosurvival factors (e.g., neurotrophins) [9]. Similar findings derive from a study in R6/1 mice, a similar genetic HD model, although with a less aggressive phenotype due to a shorter CAG repeat length in the transgene, in which authors were unable to find rotarod improvements with ∆^9^-THC, despite some benefits found in other behavioral parameters such as motor responses in exploratory tests [14].

The benefits we found in clasping behavior with the Sativex-like combination of phytocannabinoids had a biochemical correlate in the improvement of metabolic activity recorded in the basal ganglia using in vivo imaging PET analysis, as well as in different H^+^-MRS indices, potentially reflecting energy failure and neuronal deterioration. It is true that we did not observe any improvement in excitotoxicity and oxidative stress markers measured with H^+^-MRS with the Sativex-like combination of phytocannabinoids, despite the potential of this combination against both processes. This can be possibly explained by the fact that excitotoxic damage and, in particular, oxidative stress are not particularly relevant cytotoxic processes in R6/2 mice, as found here and as has been described before in other studies [9,35]. On the other hand, it would have been interesting to have data of animal survival after the treatment with the Sativex-like combination of phytocannabinoids, but this was not possible once the animals were euthanized for collecting tissues for H^+^-MRS analysis.

Collectively, the improvement in some behavioral and neurochemical parameters that have been found here in R6/2 mice after the Sativex-like combination of phytocannabinoids, together with the data obtained in neurotoxin-based models [18,20], fueled the interest in the clinical evaluation of Sativex as a disease-modifying therapy in HD patients. In fact, such clinical evaluation was initiated before our present experiment in R6/2 mice was concluded, and it was derived from the collaboration of a group of Spanish neurologists, GW Pharmaceuticals (the British pharma company that developed Sativex), and various groups of basic researchers including us. The clinical study was developed in a small population of HD patients (all of them early symptomatic), designed as a crossover trial with two different treatment patterns: (i) placebo (12 weeks), washout period (4–6 weeks), and Sativex (12 weeks); and (ii) Sativex (12 weeks), washout period (4–6 weeks) and placebo (12 weeks). The dosing of Sativex^®^/placebo was 12 sprays/day. The primary endpoint was safety of Sativex in HD patients, whereas the secondary endpoint was to obtain any evidence of slower disease progression in the patients during the active treatment phases. We observed that Sativex, in concordance with previous data obtained in control subjects, patients of other pathologies, and laboratory animals (reviewed in [36]), was safe in HD patients. However, we were unable to detect any evidence of slower disease progression [4]. It is possible that the efficacy of Sativex constituents needs periods of treatments longer than the relatively short 12 weeks used in this clinical trial. A similar situation happened with creatine, which was also a very active neuroprotective compound in preclinical studies in HD [37,38], but failed when passed to clinical validation [39]. Authors concluded that the duration of the study was the critical factor as one year, a treatment period longer than in our study with Sativex [4], was considered not sufficiently long to have clinically detectable impact in HD patients [39]. With the aim of progressing in this direction, we are presently designing a novel initiative that will attempt to recruit a higher population of HD patients, to involve different hospitals in Europe, and to work with more than one Sativex dosing regimen and longer treatment periods.

## 4. Materials and Methods

### 4.1. Animals

R6/2 and wild-type mice, produced from initial breeders obtained from Jax (Jackson Laboratories, Bar Harbor, ME, USA), were housed in rooms with controlled photoperiod (08:00–20:00 light) and temperature (22 ± 1 °C) and with free access to standard food and water. Offspring were genotyped for the transgene containing the mutated huntingtin following a procedure described previously [10]. Male R6/2 (160 ± 5 CAG repeats) and wild-type animals were used at the age of 4 weeks to record disease progression and basal ganglia deterioration in pharmacological studies using a Sativex-like combination of phytocannabinoids. All experiments were conducted according to local and European rules (directive 86/609/EEC) and approved by the “Comité de Experimentación Animal” of our university (CEA-UCM 56/2012; 8 March 2012).

### 4.2. Treatments and Sampling

Animals included in our pharmacological studies were treated with the 1:1 combination of botanical extracts enriched with either ∆^9^-THC, kindly provided by GW Research Ltd., Cambridge, UK (∆^9^-THC botanical extract contained 69.6% ∆^9^-THC, 0.3% CBD, 0.9% CBG, 0.9% cannabichromene, and 1.9% other phytocannabinoids) or CBD, also provided by GW Research Ltd., Cambridge, UK (CBD botanical extract contained 64.3% CBD, 2.3% ∆^9^-THC, 1.1% CBG, 3.0% cannabichromene, and 1.5% other phytocannabinoids). The total dose of cannabinoid administered was always 4.5 mg/kg (equivalent to 3 mg/kg of pure CBD + ∆^9^-THC), a dose within the range of effective doses of both compounds when they were administered in pure form in this and other experimental models of HD [10,11,12,13], and also close to the clinical uses of Sativex [4,36]. This was also close to the doses used in our previous studies with Sativex-like combination of phytocannabinoids in other HD models, although these studies used rats instead mice [18,20]. Cannabinoids were prepared in Tween 80-saline solution (1:16) and they were administered intraperitoneally (i.p.). R6/2 mice administered with vehicle, as well as counterpart wild-type animals, were also included in this experiment. To include an additional group of wild-type mice treated with Sativex would have been desirable. However, due to the need to consider the new 3R recommendations on the use of experimental animals and given that previous studies have reported no relevant differences between wild-type animals treated with vehicle or with different cannabinoids [9,10,11], such a group was not included in our present study. The treatment was initiated when animals were 4 weeks old (presymptomatic) and was repeated every day up to the age of 12 weeks, the age at which the animals were euthanized after behavioral (rotarod test analyzed weekly, and clasping analyzed at 10 weeks) and in vivo PET imaging (at 10 weeks) analysis. Their brains were rapidly removed and the two striata dissected and stored immediately at −80 °C for subsequent H^+^-MRS analysis. It would have been interesting to detect any possible benefit of the Sativex-like combination of phytocannabinoids on animal survival, but this would have demanded a separate experiment, which was not approved due to ethical concerns. In all experiments, at least six animals were used per experimental group.

### 4.3. Behavioral Recording

All behavioral tests were conducted prior to drug injections to avoid acute effects of the compounds under investigation, and they were carried out by researchers blinded to the treatment in each animal.

#### 4.3.1. Rotarod Test

We used a LE8200 device (Panlab, Barcelona, Spain), with acceleration from 4 to 40 rpm. over a period of 600 s. After a period of acclimation and training (first session: 0 rpm for 30 s; second and third sessions: 4 rpm for 60 s, with periods of 10 min between sessions) mice were tested on one day every week from week 4 of age, for four consecutive trials, with a rest period of approximately 20 min between trials. The average of the last three trials per day was used for the statistical analysis.

#### 4.3.2. Hindlimb Clasping

Mice were suspended by the tail, so that their body dangled in the air facing downward. Hindlimb position was observed with the mice lifted by the tail for 30 s and animals were scored according to the following scale: (i) score = 0 if the hindlimbs are consistently splayed outward, away from the abdomen; (ii) score = 1 if one hindlimb is retracted toward the abdomen; (iii) score = 2 if both hindlimbs are partially retracted toward the abdomen; and (iv) score = 3 if both hindlimbs are entirely retracted and touching the abdomen.

### 4.4. In Vivo Analysis of Glucose Metabolism: [^18^F]FDG PET Imaging

To evaluate the regional brain metabolic activity, PET with the radiotracer [^18^F]-fluoro-deoxy-glucose ([^18^F]FDG) was performed. Briefly, mice were fasted, to minimize the influence of glycemia, for at least 12 h previous to be i.p. injected with approximately 11.1 MBq of [^18^F]FDG (Instituto Tecnológico PET, Madrid, Spain). After an uptake period of 45 min, mice were scanned with a small-animal hybrid PET/CT (computed tomography) device (Albira ARS scanner, Oncovision, Valencia, Spain) under 2% isoflurane anesthesia. PET acquisition time was 30 min and it was immediately followed by CT scanning. After reconstruction of the PET and CT images, these were co-registered to a magnetic resonance image (MRI) template for the mouse brain, in which the brain regions were delineated. To this aim, the CT image was first co-registered to the MRI template and the mathematical transformation was saved. Then, this transformation was applied to its own fused PET image. This step allows the right matching of the PET image with the mouse brain MRI template. All these steps were carried out using PMOD 3.0 software (PMOD Technologies Ltd., Zurich, Switzerland). As an index of regional metabolic activity, we used the standard uptake value (SUV), which is currently the most used quantification index using [^18^F]FDG PET imaging for small animals [40]. It represents the ratio of the regional radioactivity concentration measured by the PET scanner and the actual injected dose (corrected for radiotracer decay at the time of the injection) divided by the animal weight.

### 4.5. Proton Magnetic Resonance Spectroscopy (H^+^-MRS)

Ex vivo H^+^-MRS analysis has been demonstrated to provide metabolic information with higher sensitivity and spectral resolution than in vivo magnetic resonance spectroscopy [21]. H^+^-MRS was performed in the MRI Unit of the Instituto Pluridisciplinar (Universidad Complutense, Madrid, Spain) using a Bruker AMX500 spectrometer and following previously described procedures [21,22]. The spectra generated were analyzed for Lac, NAA, Glu, GSH, Cre, Tau, and Cho, and several ratios were calculated. They are relatively predictive of excitotoxicity (Glu/NAA), energy deficits (Lac/NAA, NAA/Cho), oxidative damage (GSH/Cre), and other HD-acting neurotoxic events (Tau/Cre, Tau/NAA), all being prognostic for brain integrity [21,22,23,24,25,26].

### 4.6. Statistics

Data were assessed by one- or two-way ANOVA, followed by the Student-Newman-Keuls, Bonferroni, or Tukey tests as post-hoc assays, as required, using the GraphPad software (version 5.0, GraphPad Software, Inc., La Jolla, CA, USA).

## 5. Conclusions

In summary, in line with the promising prospects generated by its beneficial effects in neurotoxin-based models of HD, this study demonstrated that the Sativex-like combination of phytocannabinoids improved some behavioral and neurochemical parameters, but not all, related to the progression of striatal deterioration in R6/2 mice. This provides more evidence in support of its potential for developing a future disease modifying therapy for HD patients, despite the recently failed clinical evaluation of Sativex in a small population of patients. Higher dosage and longer treatments periods may be important to reveal such potentialities in patients.
